# Development of a chemically disclosed serum-free medium for mouse pluripotent stem cells

**DOI:** 10.3389/fbioe.2024.1390386

**Published:** 2024-05-15

**Authors:** Tomoka Katayama, Marina Takechi, Yamato Murata, Yuta Chigi, Shinpei Yamaguchi, Daiji Okamura

**Affiliations:** ^1^ Department of Advanced Bioscience, Faculty of Agriculture, Kindai University, Nara, Japan; ^2^ Stem Cells and Reprogramming Laboratory, Department of Biology, Faculty of Science, Toho University, Chiba, Japan; ^3^ Graduate School of Frontier Biosciences, Osaka University, Osaka, Japan; ^4^ Institute of Advanced Medical Sciences, Tokushima University, Tokushima, Japan

**Keywords:** mouse, ESCs, EpiSCs, serum-free medium, laminin, cholesterol, naïve and primed states

## Abstract

Mouse embryonic stem cells (mESCs) have been widely used as a model system to study the basic biology of pluripotency and to develop cell-based therapies. Traditionally, mESCs have been cultured in a medium supplemented with fetal bovine serum (FBS). However, serum with its inconsistent chemical composition has been problematic for reproducibility and for studying the role of specific components. While some serum-free media have been reported, these media contain commercial additives whose detailed components have not been disclosed. Recently, we developed a serum-free medium, DA-X medium, which can maintain a wide variety of adherent cancer lines. In this study, we modified the DA-X medium and established a novel serum-free condition for both naïve mESCs in which all components are chemically defined and disclosed (DA-X-modified medium for robust growth of pluripotent stem cells: DARP medium). The DARP medium fully supports the normal transcriptome and differentiation potential in teratoma and the establishment of mESCs from blastocysts that retain the developmental potential in all three germ layers, including germ cells in chimeric embryos. Utility of chemically defined DA-X medium for primed mouse epiblast stem cells (mEpiSCs) revealed that an optimal amount of cholesterol is required for the robust growth of naïve-state mESCs, but is dispensable for the maintenance of primed-state mEpiSCs. Thus, this study provides reliable and reproducible culture methods to investigate the role of specific components regulating self-renewal and pluripotency in a wide range of pluripotent states.

## Introduction

Embryonic stem cells (ESCs), derived from the inner cell mass of embryos at the blastocyst stage, have the ability to self-renew and retain the capacity to differentiate into all three germ layers (Martin, 1981; Evans and Kaufman, 1981; Bradley et al., 1984). Historically, mouse ESCs (mESCs) have been established and maintained in the medium supplemented with fetal bovine serum (FBS) along with leukaemia inhibitory factor (LIF) and mouse embryonic fibroblasts (MEFs) (Ohtsuka et al., 2015). FBS contains over 1,000 different components, including proteins, carbohydrates, growth factors, lipids, etc. (Shah, 1999; Even et al., 2006) LIF is a key cytokine for maintaining the pluripotency of mESCs, and MEFs play a key role in the culture of mESCs, acting as feeder cells to provide a supportive microenvironment for mESC self-renewal (Williams et al., 1988; Smith et al., 1988). However, FBS containing media have potential problems, including contamination with animal-derived factors, batch-to-batch variability, and the difficulty in controlling the culture environment (van der Valk et al., 2010). For these reasons, many researchers have recently turned to the use of serum-free and feeder-free condition for mESC culture.

Several studies have reported on chemically defined serum-free media for mESC maintenance (Ying et al., 2003; Nichols and Ying, 2006; Ying et al., 2008; Hayashi et al., 2008; Konishi et al., 2018), and the introduction of “2i culture conditions” using two small molecule inhibitors, PD0325901 and CHIR99021, marked a significant advance in these serum-free culture systems (Ying et al., 2008). Currently, the most commonly used serum-free media contain Knock-out Serum Replacement (KSR, Thermo Fisher Scientific) or B-27 supplement (Thermo Fisher Scientific) as a replacement for FBS (Ying et al., 2003; Ying et al., 2008). However, since KSR and B-27 supplement are commercially available products, their detailed ingredients and concentrations are not disclosed.

Pluripotent stem cells exist in two major states. The naïve state, which corresponds to cells in the inner cell mass in early embryogenesis (pre-implantation), is represented by mESCs, whereas the primed state, which is similar to epiblast cells after implantation, is represented by mouse Epiblast Stem Cells (mEpiSCs) and human ES/iPS cells (Nichols and Smith, 2009). In addition, there are “ground states,” which are thought to capture the most extreme pluripotent states of naïve and primed; the naïve ground state is captured by the culture conditions with the addition of 2i, whereas the primed ground state is captured by the addition of FGF2 and Wnt inhibitors (Sumi et al., 2013; Sugimoto et al., 2015), which is also the case for region selective EpiSCs (rsEpiSCs) (Wu et al., 2015). Thus, pluripotent stem cells are useful for studying the biological properties of early embryonic cells because they mimic different developmental stages including ground states and can reproduce the signals and microenvironment required for each stage. However, in previous studies, the use of media containing supplements with undisclosed components, such as KSR and B-27 supplement, has limited our understanding of stem cell requirements for specific components and their role in stem cell properties such as self-renewal. A more detailed understanding and optimization of media components would contribute to the advancement of stem cell research, increasing its credibility and range of applications.

Recently, we developed a defined serum-free culture condition consisting of Dulbecco’s Modified Eagle’s Medium (DMEM) as the basal medium supplemented with albumin (BSA) and insulin-transferrin-selenium-ethanolamine (ITS-X) (hereafter referred to as “DA-X medium”). DA-X medium successfully maintained a variety of different types of adherent cancer cell lines, including HeLa, MCF-7 and A549, etc., with survival and proliferation comparable to those in a conventional serum-containing medium (Takii et al., 2022). In this study, we took advantage of the DA-X medium and used it to develop a DA-X-modified medium for robust growth of pluripotent stem cells (DARP medium). During the development, we identified the essential and minimal components required for the growth and maintenance of mESCs and an optimal concentration of cholesterol supplementation that promoted mESC proliferation in long-term culture, the growth of which reached comparable level to that in KSR-supplemented medium. We also demonstrated that mESC lines could be successfully established from blastocysts using the DARP medium and that they exhibited developmental potential, including primordial germ cells in the chimera embryos. Furthermore, the DA-X-modified medium was also able to support the undifferentiated state of primed mEpiSCs. In sharp contrast to mESCs, cholesterol had no beneficial effect on maintaining mEpiSCs.

## Materials and methods

### Mouse ESCs culture with conventional chemically-defined media

Mouse embryonic fibroblast (MEF) feeder cells were prepared from E14.5 mouse embryos. E14tg2a mESC line derived from 129/Ola (Riken BRC, AES0135) (Hooper et al., 1987) and R26-WntVis mESC line, a modified Rosa26 locus that contains a Wnt-responsive reporter gene (Takemoto et al., 2016), were maintained on mitomycin C (MMC) (FUJIFILM, 139-18711)-inactivated MEF feeder cells on single well of 4-well plate (SPL Life Sciences, SPL-30004) in DF10^2iLIF^ medium (described in Supplementary Table S1). The medium was changed every 2 days, and then colony-forming mESCs were dissociated by TrypLE Express Enzyme (Gibco, 12604-013) and passed onto newly prepared MMC-inactivated MEF feeder cells for further cultivation at a split ratio of 1:20 every 4-5 days. For switching to each serum-free defined medium from DF10^2iLIF^ medium, 1.5 × 10^5^ cells were seeded with each medium onto MMC-inactivated MEF feeder cells or extra-cellular matrix-coated plate. The detailed compositions of the media are described in the Supplementary Table S1. The information of extra-cellular matrix is described below. Prior to seeding mESCs and rsEpiSCs, 4-well cell culture plates were pre-coated with the extracellular matrixes for 1 h at 37°C at the concentration of 2.35 μg/cm^2^ for Fibronectin (Sigma-Aldrich., F1141), 1.25 μg/cm^2^ for Vitronectin (FUJIFILM, 220–02041), 0.5 μg/cm^2^ for recombinant Laminin511-E8 fragments (iMatrix-511, MAX, 892011), 2% (v/v) for Matrigel Matrix (Corning, 354234), 0.2% (w/v) for Gelatin (Sigma, G1890), and 0.2% (w/v) for Collagen Type Ⅰ (FUJIFILM, ASC-1-100). And incubated, and then aspirated for use. To optimize the laminin concentration, the laminin solution was diluted with PBS for use at indicated concentration in Supplementary Figure S1.

### DA-X and DARP medium preparation

The detailed compositions of DA-X and DARP medium are described in the Supplementary Table S1. In optimization of cholesterol concentration for better proliferation of mESCs, soluble cholesterol was administrated at a range between 5 and 25 µM. Depending on the experiments with DA-X medium, 2i or 2iLIF were supplemented. All media components including 2i and LIF were pre-mixed for preparation, but water-soluble cholesterol solution was added to the medium just before use to prevent crystallization in storage at 4°C. To enhance cell viability during the resuscitation process of cryopreserved cells cultured in DARP medium, employing pre-warmed serum containing DF10 medium for washing out the cryopreservation solution yielded improved results.

### Preparation of water-soluble cholesterol solution

For the preparation of 50 mM cholesterol/EtOH stock solution, 19.3 mg of cholesterol (Sigma, C8667) was dissolved in 1 mL of 100% EtOH by incubation at 80°C. For water-solubilization of the cholesterol/EtOH solution, 42 mg of Methyl-β-cyclodextrin (Sigma-Aldrich, 332615) was added to the 1 mL of 5 mM cholesterol/EtOH solution. The solution was stored at −30°C before use.

### Mouse rsEpiSCs culture

The mouse rsEpiSC (region-selective Epiblast Stem Cell) line was established from E6.25 epiblast (ICR strain background) (Okamura et al., 2021). The rsEpiSC was maintained on MMC-inactivated MEF feeder cells on single well of 4-well plate in N2B27^FR^ (described in Supplementary Table S1). The medium was changed every day, and then colony-forming rsEpiSCs were dissociated by TrypLE Express Enzyme and passed onto newly prepared MMC-inactivated MEF feeder cells for further cultivation at a split ratio of 1:20 every 3-4 days. For switching of base media, 2.5 × 10^5^ cells were seeded with DA-X^FR/β−ME^ (described in Supplementary Table S1) onto MMC-inactivated MEF feeder cells or Laminin-coated plate. Depending on the experiments, cholesterol was added into DA-X^FR/β-ME^ medium. For resuscitation of cryopreserved cells cultured in DA-X^FR/β-ME^, pre-warmed serum-containing DF10 medium for washing out the cryopreservation solution improved cell viability.

### Alkaline phosphatase (ALP) staining

Cultured cells were fixed with 4% (w/v) paraformaldehyde in PBS for 15 min at room temperature. The cells were washed three times in PBS and then stained with the ALP staining solution. Preparation of ALP staining solution was as followins; 5 mg of naphthol AS-MX phosphate disodium salt (Sigma, N5000) was dissolved in 500 µL of N, N-Dimethylformamide, and then 10 mg of Fast Blue RR salt (Wako, 069-03712) was dissolved in the above solution. Next, the solution was added to 9.5 mL of 0.1 M Tris-HCl (pH 9.5) and the resulting staining solution was filtrered through a 0.2 µm filter to remove precipitates. The staining solution was then incubated with fixed cells for more than 10 min at room temperature for better development. Stained cells were then washed three times in PBS and filled with PBS for observation under a microscope (Keyence, BZ-X710).

### Western blotting protocol

Briefly, proteins from cell lysates were separated on 12% (w/v) SDS-PAGE and transferred onto a PVDF membrane (Millipore, IPVH00010). The membrane was blocked in 0.3% (w/v) nonfat dry milk, TBST (1x TBS, 0.1% Tween-20) as blocking buffer, and then incubated with the primary antibody in Can Get Signal Immunoreaction Enhancer Solution I (TOYOBO, NKB201) as diluent at 4°C overnight. After washing with TBST, the membrane was incubated with the horseradish peroxidase-conjugated secondary antibody in Can Get Signal Immunoreaction Enhancer Solution II (TOYOBO, NKB2301) as diluent at room temperature overnight. Bands were visualized using a chemiluminescent detection system (ATTO, WSE-6100H-CP LuminoGraph I). Primary antibodies used in this study are listed below: anti-AKT antibody (B-1) (diluted at 1:1000, Santa Cruz, sc-5298), anti-phospho AKT1/2/3 antibody (11E6) (diluted at 1:1000, Santa Cruz, sc-81433), anti-STAT3 antibody (D3Z2G) (diluted at 1:5000, Cell Signaling Technology, 12640S), anti-phospho STAT3 antibody (D3A7) (diluted at 1:5000, Cell Signaling Technology, 9145S), anti-β-Actin antibody (C-4) (diluted at 1:5000, Santa Cruz, sc-47778). Secondary antibodies used in this study are listed below: anti-IgG (H + L chain) (Mouse) pAb-HRP (diluted at 1:10000, MBL, 330), anti-IgG (H + L chain) (Rabbit) pAb-HRP (diluted at 1:10000, MBL, 458).

### Teratoma formation

mESCs or rsEpiSCs at 2 × 10^6^ cells were resuspended in a 1:1 mixture of 100 µL of ice-cold PBS with 100 µL of Matrigel (Corning, 354234) and injected subcutaneously into male immunodeficient nude mice (KSN/Slc) at 8–16 weeks of age to form teratomas. After 4 weeks, the mice were sacrificed by cervical dislocation, and teratomas were excised, fixed in 4% (w/v) paraformaldehyde in PBS overnight at 4°C, followed by incubation in 10% (w/v) sucrose/PBS overnight at 4°C and then 20% (w/v) sucrose/PBS overnight at 4°C. Tissue samples were embedded in OCT (Tissue-Tek) for cryo-section at 10 μm, and stained with hematoxylin-eosin or used for immunofluorescence.

### Karyotypic analysis

To evaluate the karyotype of mESCs, metaphase spreads were prepared as described in previous study (Takagi et al., 1983). Briefly, undifferentiated mESCs were treated with 10 μg/mL KaryoMax colcemid solution (Invitrogen) for 1 h and dissociated into single cells using TrypLE Express Enzyme. After hypotonic treatment with 75 mM KCl solution for 10 min at room temperature, mESCs were fixed in Carnoy’s solution (methanol/acetic acid, 3:1). Chromosome spreads were prepared by an air-drying method (Takagi et al., 1983) on APS-coated adhesive glass slides (MATSUNAMI, APS-01) and stained for 10 min with a Giemsa’s Stain Solution for Microscope (Giemsa’s Azure Eosin Methylene Blue solution, Merck Millipore, 1.09204.0103). Stained chromosomes were mounted with a Permount mounting medium (Fisher Scientific, SP15-100). At least, 140 metaphase nuclei were examined for each culture condition.

### Immunohistochemistry

Cells grown on a 4-well plate were fixed with freshly prepared 4% (w/v) paraformaldehyde in PBS for 15 min at room temperature, permeabilized with 1% (v/v) Triton-X, 1% (w/v) bovine serum albumin (BSA), and blocked with 10% (v/v) FBS-contained PBS as blocking buffer for 1 h at room temperature. The cells were incubated with primary antibodies in 10% (v/v) FBS, 1% (w/v) BSA, and 1% (v/v) Triton-X-containing PBS as diluent overnight at 4°C. On the following day, the cells were washed three times in 0.1% (v/v) Triton/PBS and incubated with fluorescent-conjugated IgG (H + L) secondary antibodies for immunofluorescence analyses at 1:500 dilutions in 1% (v/v) FBS, 1% (w/v) BSA, and 1% (v/v) Triton-X-containing PBS as diluent for 2 h at room temperature. The cells were washed three times and nuclei were counter-stained with DAPI (Sigma-Aldrich, D9542). Primary antibodies used in this study include anti-OCT4 antibody (C-10) (diluted at 1:300, Santa Cruz, sc-5279), anti-NANOG antibody (diluted at 1:100, Santa Cruz, sc-29312), anti-SOX2 antibody (E-4) (diluted at 1:300, Santa Cruz, sc-365823), anti-OTX2 antibody (diluted at 1:200, R&D systems, AF 1979), anti-β-Tubulin III antibody (diluted at 1:1000, Sigma-Aldrich, T2200), anti-SMA/Actin/α-Smooth Muscle antibody (diluted at 1:600, Sigma-Aldrich, A5228), anti-AFP/Alpha-fetoprotein antibody (C3) (diluted at 1:300, Santa Cruz, sc-8399), anti-TRA98 antibody (diluted at 1:1000, BioAcademia, 73-003), anti-GFP antibody (diluted at 1:500, Invitrogen, A11122). Secondary antibodies used in this study are listed below: for immunofluorescence analyses, goat anti-mouse (Alexa Fluor 488, abcam, ab150113), (Alexa Fluor 594, abcam, ab150116), goat anti-rabbit (Alexa Fluor 594, abcam, ab150080), donkey anti-goat (Alexa Fluor 594, abcam, ab150132).

**FIGURE 1:**
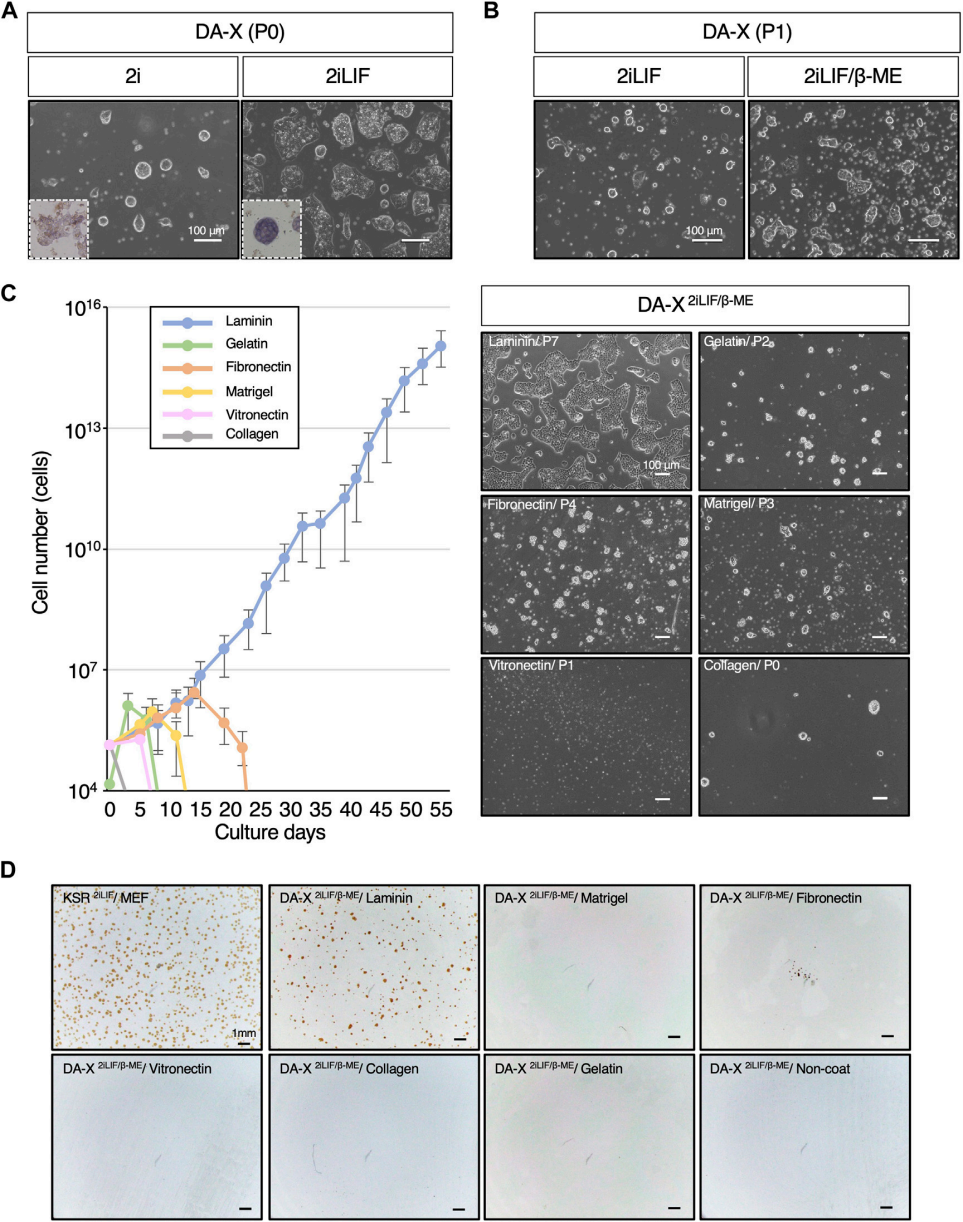
Laminin exclusively supports the proliferation of mESCs in DA-X-based serum-free medium with 2iLIF. **(A,B)** Representative images of mouse Embryonic Stem Cells (mESCs) under indicated medium conditions on fibronectin. The number of passages was counted after passage from the DF10^2iLIF^ medium condition. **(A)** Images were captured at passage 0 (P0) on day 5 in DA-X^2i^ and on day 2 in DA-X^2iLIF^. Square dotted lines in the images represent mESC colonies stained for alkaline phosphatase activity at higher magnification. **(B)** Images were captured in DA-X^2iLIF^ and DA-X^2iLIF/β−ME^ on day 2 of passage 1 (P1). **(C)** The growth curves on different ECMs for 55 days and the representative images of mESC colonies cultured on day 3 of the indicated passage number. Error bars indicate s.d. (*n* = 3, biological replicates). **(D)** Analysis of single cell cloning efficiency on different ECMs. 1.0 × 10^4^ dissociated mESCs were seeded in KSR^2iLIF^ on MEF feeder cells or in DA-X^2iLIF/β-ME^ medium on the indicated ECMs. Cloning efficiency was measured by the number of OCT4-positive colonies at day 4.

For the cloning efficiency assay shown in Figure 1D, OCT4 protein expression was detected by DAB staining. All procedures were similar to the immunofluorescence analysis described above, except that anti-OCT4 antibody (C-10) (diluted at 1:300, Santa Cruz, sc-5279) was used as primary antibody and horseradish peroxidase-conjugated IgG (H + L) antibodies (diluted at 1:500, MBL, 330) was used as secondary antibody. OCT4 expression was visualized by the peroxidase stain DAB kit (brown stain) (nacalai tesque, 25985-50) and observed under a microscope (Keyence, BZ-X710).

### RNA preparation and reverse transcription real-time PCR

Total RNA was extracted using RNeasy Mini Kit (QIAGEN, 74104) according to the manufacturer’s instructions. RNAs were reverse transcribed using ReverTra Ace qPCR RT Master Mix (TOYOBO, FSQ-201), and real-time PCR was performed using THUNDERBIRD SYBR qPCR Mix (TOYOBO, QPS-201) in MIC qPCR (bio molecular systems). The relative expression levels of each gene were normalized by Gapdh and calculated by the comparative CT method. Primer sequences are listed below: *Gapdh*-F (5′-AGG​TCG​GTG​TGA​ACG​GAT​TTG-3′), *Gapdh*-R (5′-TGT​AGA​CCA​TGT​AGT​TGA​GGT​CA-3′), *EGFP*-F (5′-CCA​CAT​GAA​GCA​GCA​GGA​CTT-3′), *EGFP*-R (5′-GGT​GCG​CTC​CTG​GAC​GTA-3′), *Oct4*-F (5′-GAT​GCT​GTG​AGC​CAA​GGC​AAG-3′), *Oct4*-R (5′-GGC​TCC​TGA​TCA​ACA​GCA​TCA​C-3′), *Sox2*-F (5′-GCG​GAG​TGG​AAA​CTT​TTG​TCC-3′), *Sox2*-R (5′-CGG​GAA​GCG​TGT​ACT​TAT​CCT​T-3′), *Nanog*-F (5′-CTT​TCA​CCT​ATT​AAG​GTG​CTT​GC-3′), *Nanog*-R (5′-TGG​CAT​CGG​TTC​ATC​ATG​GTA​C-3′).

### RNA-sequencing and data analysis

Sequencing libraries were prepared using the NEBNext Ultra II Directional RNA Library Prep Kit (NEB) and were sequenced on an Illumina Novaseq platform to generate 150 bp paired-end reads. The adaptors of the obtained raw sequence data were trimmed using Trim Galore (v0.6.3), and quality control using FastQC (v0.11.7). Processed reads were aligned to the mouse reference genome (UCSC mm10) using STAR (v2.7.0c) (Dobin et al., 2013). FeatureCounts v1.5.2 was used to generate the read-counting data for each gene. Differential gene expression analysis was performed using TCC (Sun et al., 2013; Tang et al., 2015). MDS plot analysis and Heatmap clustering were performed using the princomp, and heatmap packages from R.

### Animals

F1 mice from C57BL/6NCrSlc x DBA/1JJmsSlc crossing (B6D2F1), ICR, and vasectomy ICR were obtained from Sankyo-lab, Japan. Male R26GRR (C57BL/6N) mice (Hasegawa et al., 2013) were used for chimera experiments.

### Derivation of DARP-ESC line from blastocysts

Female B6D2F1 mice were injected with 100 µL of CARD HyperOva (KYUDO, Japan). After 48 h, they were injected with 7.5 IU of human chorionic gonadotropin (hCG, Asuka pharma) and mated with male R26GRR mice (Hasegawa et al., 2013). The appearance of the vaginal plug at noon was defined as embryonic day (E) 0.5. On E3.5 embryos were collected by flushing the oviduct or uterus with M2 media containing 4 mg/mL BSA. The zona pellucidae were then removed using 0.5% (w/v) pronase (Merck). The embryos were placed in single wells of 96-well plates coated with 0.2% (w/v) gelatin/PBS and mouse embryonic fibroblasts (MEFs) as feeder cells and cultured under DARP medium. To adapt the established DARP-ESCs to feeder-free conditions, TrypLE Select Enzyme (Gibco, 12563011) was washed out with 10% FBS containing DARP medium after dissociation and centrifuged to pellet before seeding onto Laminin-coated plates with DARP medium.

### Creating chimeric embryo

Female B6D2F1 mice were injected with 100 µL of CARD HyperOva (Kyodo, Japan). After 48 h, they were injected with 300 µL of hCG and mated with male B6D2F1 mice. On E2.5, embryos were collected by flushing of oviduct or uterus with M2 media containing 4 mg/mL BSA. The zona pellucidae were removed using 0.5% (w/v) pronase. The embryos were placed in each aggregation hole with KSOM, and three DARP-ESCs were added. The aggregated E4.5 embryos were then transferred to the uterus of pseudo-pregnant ICR mice.

### Statistical analysis

Statistical analysis was performed using one-way ANOVA, when significant, group differences were evaluated using a Student’s t-test. p values < 0.05 were considered as statistically.

## Results

### DA-X medium supports mESC survival and proliferation with 2iLIF and β-mercaptoethanol

To ascertain the capacity of DA-X medium to support not only adherent cancer cell lines but also mouse pluripotent stem cell lines, mESCs were maintained in DA-X medium on fibronectin-coated plates, which is one of the most efficient extracellular matrices for robust growth of adherent cancer cell lines (Cao et al., 2015). In order to minimize the composition of the medium, an initial evaluation was performed with DA-X medium containing 2i (Ying et al., 2008; Williams et al., 1988; Smith et al., 1988), and then the missing components were stepwise modified or added one by one. As 2i was previously reported to be sufficient for mESC self-renewal (Nichols and Smith, 2009), mESCs in DA-X medium with 2i (DA-X^2i^) showed representative dome-shaped colony morphology in the undifferentiated state (Figure 1A), but weak signals on alkaline phosphatase (ALP) staining, indicating partial differentiation of mESCs (Figure 1A). Although the intensity of ALP staining and growth were improved in DA-X medium with 2iLIF (DA-X^2iLIF^) (Figure 1A), cell viability and proliferation could not be maintained even after a single passage (Figure 1B). Supplementation with β-mercaptoethanol (β-ME), an antioxidant widely used in the culture of pluripotent stem cells since the first derivation of mESCs (Martin, 1981; Evans and Kaufman, 1981) greatly improved the survival and proliferation. The mESCs in DA-X medium with 2iLIF/β-ME (DA-X^2iLIF/β−ME^) exhibited a dome-shaped colony morphology indicative of a typical undifferentiated state, and exponential growth was also maintained over multiple passages of culture (Figure 1B).

### Laminin supports the proliferation of mESCs in DA-X medium-based with 2iLIF

The extracellular matrix (ECM) plays a pivotal role in governing cell proliferation and differentiation, not only *in vivo* but also *in vitro* conditions. To identify the specific ECM capable of supporting mESC proliferation and cell viability under DA-X^2iLIF/β−ME^ conditions better than fibronectin, we systematically tested laminin, gelatin, fibronectin, matrigel, vitronectin, and collagen type I, which are commonly used in feeder-free culture of ESCs/iPSCs (Watt and Huck, 2013). Among these ECMs, we found that only laminin coating supported continuous proliferation of mESC (Figure 1C). A unique monolayered, amoeboid-like colony morphology of mESC was well maintained in the laminin-coated condition over serial passages of more than 55 days (Figure 1C). In contrast, none of the other ECMs showed comparable proliferation and viability of mESCs. The concentration of laminin appears to be important, since Matrigel, despite having laminin as its main composition (manufacturer’s information), did not support in the robust proliferation of mESCs. The laminin concentration used in this study was recommended by the manufacturer, and indeed, was found to be the most suitable concentration for the survival and proliferation of mESCs (Supplementary Figure S1).

The expansion of single-cell mESCs with OCT4-positive undifferentiated colonies was also only achieved under laminin-coated conditions (Figure 1D). However the efficiency of clonal expansion of mESCs in KSR-contained medium with 2iLIF (KSR^2iLIF^) on the feeder layer was slightly better than that in DA-X^2iLIF/β-ME^ conditions. These results indicate that DA-X medium is not only compatible with adherent cancer cell lines but also with undifferentiated mESCs on laminin-coated condition (Figure 1).

### Cholesterol enhances the proliferation of mESCs in DA-X-modified medium

**FIGURE 2:**
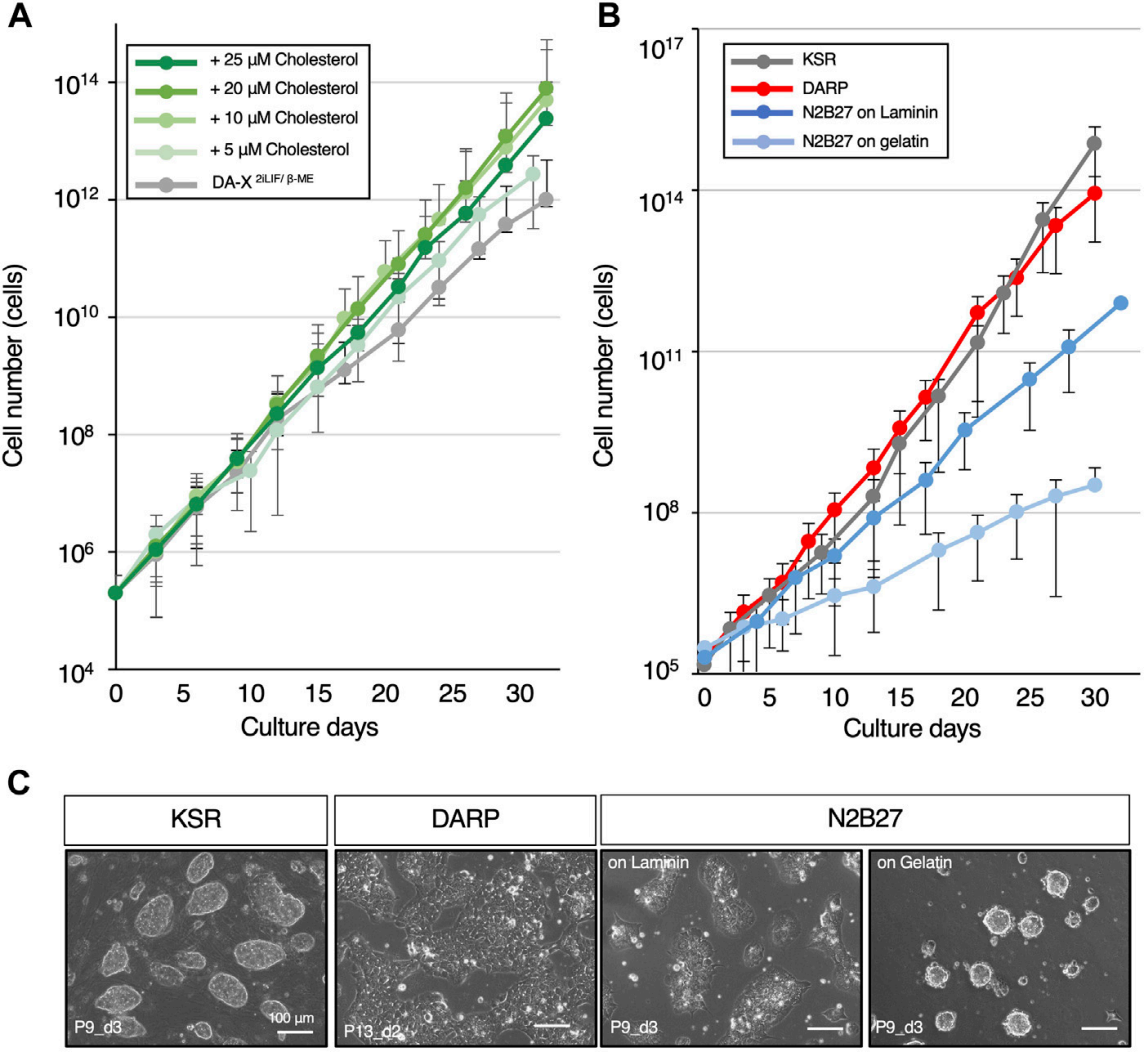
DARP medium containing cholesterol achieves the robust growth of mESCs. **(A)** Optimization of cholesterol concentration for mESC growth in DA-X^2iLIF/β-ME^ medium condition. The growth-promoting effect of cholesterol on mESCs was observed up to 20 μM. **(B)** The growth curves for comparison of mESC growth between the serum-free defined media conditions. **(C)** Representative images of mESC colony morphology under the growth comparison in Figure 2B. Images were captured at the indicated passages and days (P9_d3 means that the image was captured on day 3 of passage 9). Error bars indicate s.d. (*n* = 3, biological replicates).

In mammalian cells, cholesterol plays a pivotal role in various cellular processes, such as membrane fluidity and cell signaling, in a concentration-dependent manner (van Aalst and Wylie, 2021; Goddard and Watts, 2012; Luchetti et al., 2016). In addition, modulations of cholesterol levels in the culture medium are known to affect cell growth across diverse cell types (Maja and Tyteca, 2022; Oguro, 2019). Nevertheless, the specific interplay between cholesterol and mouse pluripotent stem cells remains unexplored. The primary reason is that conventional medium contained FBS, which is rich in cholesterol, and it was technically challenging to specifically remove cholesterol from it. Culture conditions without serum, such as N2B27-based medium, are unsuitable for this kind of analysis because their precise compositions are unknown. Our chemically defined and disclosed DA-X-modified medium enabled us to investigate whether cholesterol played a role in cellular processes in mESCs. Proliferation at early passages was unaffected by cholesterol in DA-X^2iLIF/β-ME^, however, the presence of cholesterol promoted the proliferation of mESCs at late passages in a cholesterol concentration-dependent manner (Figure 2A). After 30 days in culture, 10 µM cholesterol-treated mESCs displayed an approximately 100-fold higher proliferation rate than cholesterol-free mESCs. These results indicated that cholesterol dose-dependently enhanced long-term mESC culture under serum-free media conditions and that the DA-X^2iLIF/β-ME^ is therefore suitable for analyzing the metabolic requirements and mechanism in mESCs. The DA-X^2iLIF/β-ME^ medium containing 10 µM cholesterol is hereafter referred to as DARP medium (DA-X-modified medium for robust growth of pluripotent stem cells).

Several studies have highlighted the importance of lipid rafts in the proliferation and self-renewal of mESCs (Lee et al., 2010; Karouzaki et al., 2021). For effective LIF signaling transduction, LIF signaling receptors, LIFR and gp130, must be localized in lipid rafts, and cholesterol level in the cell membrane influences the lipid raft-dependent signal intensity (Nishihara, 2018). Due to the crucial role played by the PI3K-AKT pathway and STAT3 in LIF signaling in mESC proliferation, we asked whether cholesterol in DARP might affect the phosphorylation of AKT and STAT3 (Niwa et al., 2009; Takahashi et al., 2005; Niwa et al., 1998; Matsuda et al., 1999). However, the phosphorylated forms of AKT and STAT3 proteins were not affected under cholesterol-contained condition (Supplementary Figure S2A).

In light of the association of high cholesterol levels and increased Wnt pathway activity in cancer cells, we next examined if cholesterol levels influenced the activation of the Wnt signaling pathway, which is critical for maintaining self-renewal and pluripotency in mouse ESCs (Kielman et al., 2002; Ogawa et al., 2006; Sheng et al., 2014). We utilized the R26-WntVis mESC line, in which the activity of Wnt signaling pathway can be monitored by the EGFP expression (Takemoto et al., 2016). However, no detectable change in protein and transcript levels of the Wnt-EGFP signaling was observed between cholesterol- or cholesterol-free mESCs (Supplementary Figure S2B). Taken together, these data suggest that alternative mechanisms are likely to drive the proliferation of mESCs in the presence of cholesterol.

In comparison between DARP medium and commonly used serum-free culture conditions for mESCs supplemented with 2iLIF (KSR and N2B27), the growth curves were examined through serial passages over 30 days (Figure 2B). The results revealed that the proliferative capacity in DARP medium was comparable to that in KSR and significantly superior to that in N2B27 medium, even on laminin-coated plates. As a characteristic of mESCs maintained in DARP medium, it was observed that the colony morphology of mESCs was still unique even with cholesterol supplementation, presenting a monolayered, amoeboid-like shape rather than the conventional dome-shaped form (Figure 2C). Taken together, DARP medium with its distinctive colony morphology achieved robust mESC growth comparable to or even better than other conventional serum-free conditions.

In addition, non-essential amino acids solution (NEAA) is a common substance supplemented to mESC cultures for survival and proliferation in both serum-containing and chemically defined media (Zhang and Feng, 2022), but when added to DARP medium, no significant positive effects on the growth were observed (Supplementary Figure S3).

### DARP medium for the maintenance of mESC pluripotency

**FIGURE 3:**
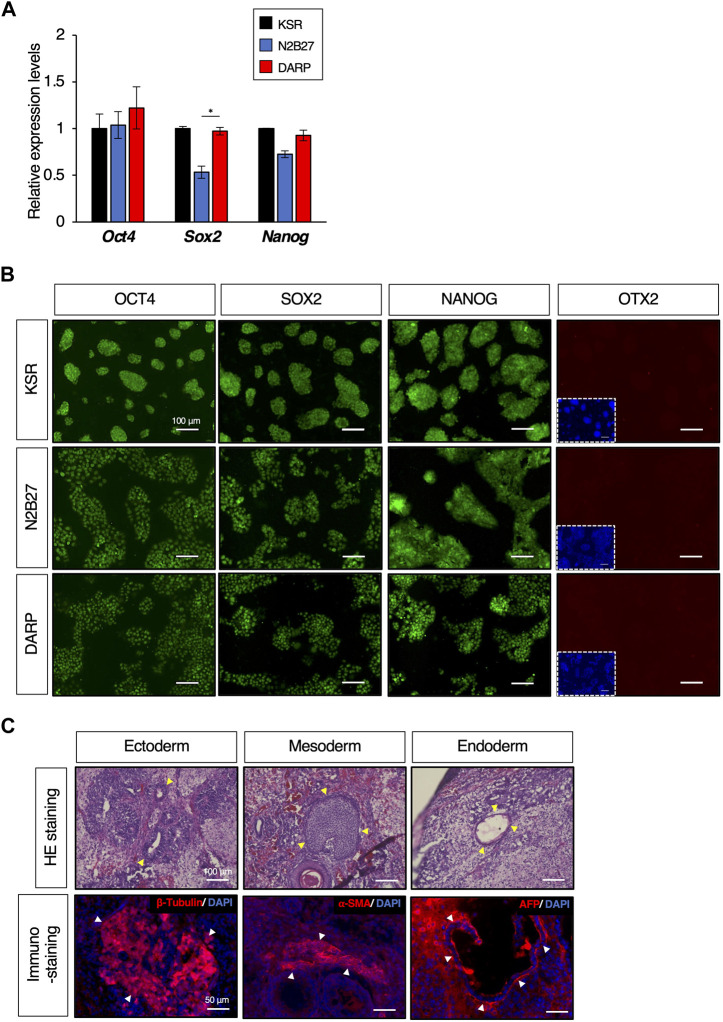
DARP medium for the maintenance of mESC naïve pluripotency. **(A)** Quantitative PCR analysis of expression of pluripotent markers of *Oct4*, *Sox2*, and *Nanog* in growing mESCs cultured in KSR, N2B27, DARP medium. Error bars indicate s.d. (*n* = 4, biological replicates). *t*-test, *p < 0.05. **(B)** Immunofluorescence to detect the expression of OCT4, SOX2, and NANOG proteins and the absence of OTX2 expression (a marker for primed state protein expression). Images were captured on day 3 of passage 6. **(C)** Histological analysis by HE stains and immunostaining with sections from teratomas generated in nude mice from mESCs cultured in DARP^2iLIF^ medium. The yellow-filled arrowheads show distinctive organizational tissues for neuroepithelium (ectoderm, top left), cartilage (mesoderm, top middle), and gland (endoderm, top right). The white-filled arrowheads indicate expression of characteristic marker protein, β-Tubulin for neuroepithelium (Bottom left), α-SMA for smooth muscle (Bottom middle), and AFP for glands (Bottom right). Nuclei were counterstained with DAPI.

To determine whether the DARP medium maintained the undifferentiated state of the mESCs, we next examined the expression of pluripotent markers. The mESCs maintained in KSR, N2B27, and DARP conditions exhibited comparable expression of pluripotent genes at both mRNA and protein levels, as depicted in Figures 3A, B, respectively. In contrast, the primed state marker protein OTX2 was not detectable in any of the mESCs, suggesting that mESCs maintained in DARP medium remain in a naïve state (Figure 3B). Chromosomal stability was confirmed by karyotypic analysis of mESCs cultured in DARP medium over prolonged periods at more than 20 passages (Supplementary Figure S4) (Abranches et al., 2013). The differentiation potential of mESCs in DARP medium into three germ layers was confirmed *in vivo* by teratoma formation (Figure 3C). Successful differentiation into neuroepithelium (ectoderm), cartilage (mesoderm), smooth muscle (mesoderm), and glands (endoderm) was confirmed by the histological analysis of the resulting teratomas.

### Gene expression profiles of mESCs in DARP as a naïve state of pluripotency

**FIGURE 4:**
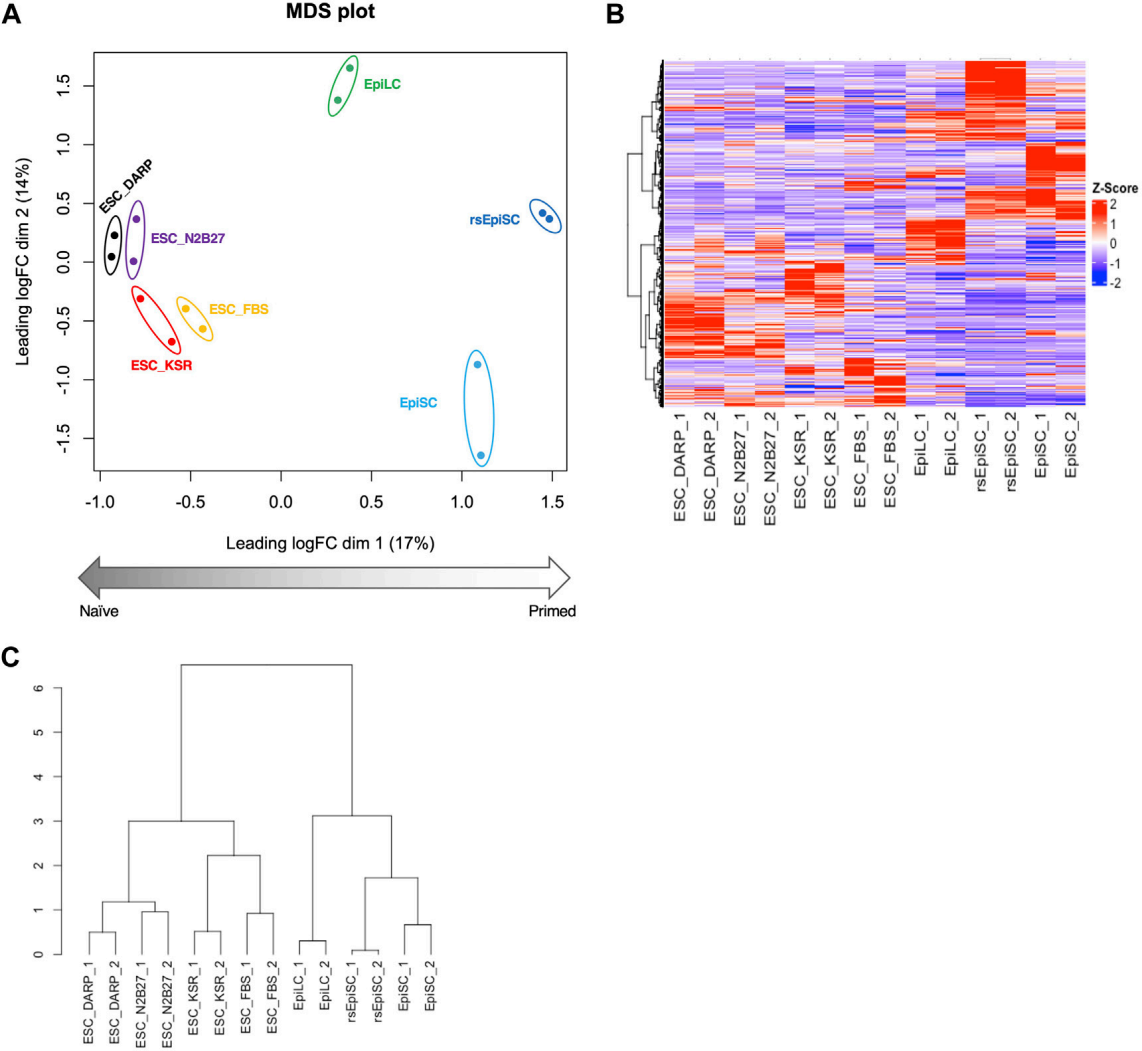
Analysis of the gene expression profiles of mESCs in DARP as a naïve state of pluripotency. Bioinformatic analysis of RNA-sequencing data with mouse pluripotent stem cells including ESCs cultured in each condition. **(A)** MDS (Multidimensional scaling) plot of each sample shows distinct clustering according to their pluripotent states, naïve to primed. Replicates of the same cell lines and conditions are depicted with colored circles, ESC_DARP (black), ESC_N2B27 (purple), ESC_KSR (red), ESC_FBS (yellow), EpiLC (green, published data set (Shirane et al., 2016)), EpiSC (light blue, published data set (Wu et al., 2015)), and rsEpiSC (blue, published data set (Wu et al., 2015)). **(B,C)** Hierarchical clustering heatmap **(B)** and hierarchical clustering dendrogram under optimal rank **(C)** based on differentially expressed genes (DEGs) among seven different cultured conditions and cell types. The number after the sample name indicates the biological replicates. Z-scaled expression levels of genes were illustrated in a heatmap.

The transcriptome of mESCs in different culture conditions was analysed by RNA-sequencing to provide a global view of the undifferentiated state. Principal component analysis (PCA) of the transcriptome MDS plot (Figure 4A), heatmap (Figure 4B) and hierarchical clustering dendrogram (Figure 4C) based on differentially expressed genes (DEGs) revealed that the gene expression profiles of mESCs in DARP medium (ESC_DARP) showed a closer clustering with those of the mESCs, naïve pluripotency, maintained in N2B27 medium (ESC_N2B27), in KSR medium (ESC_KSR), in FBS medium (ESC_FBS), while showing a clear separation from EpiLCs, formative pluripotency, EpiSCs and rsEpiSCs, primed pluripotency.

### Germline competence of mESCs established in DARP medium in chimera

**FIGURE 5:**
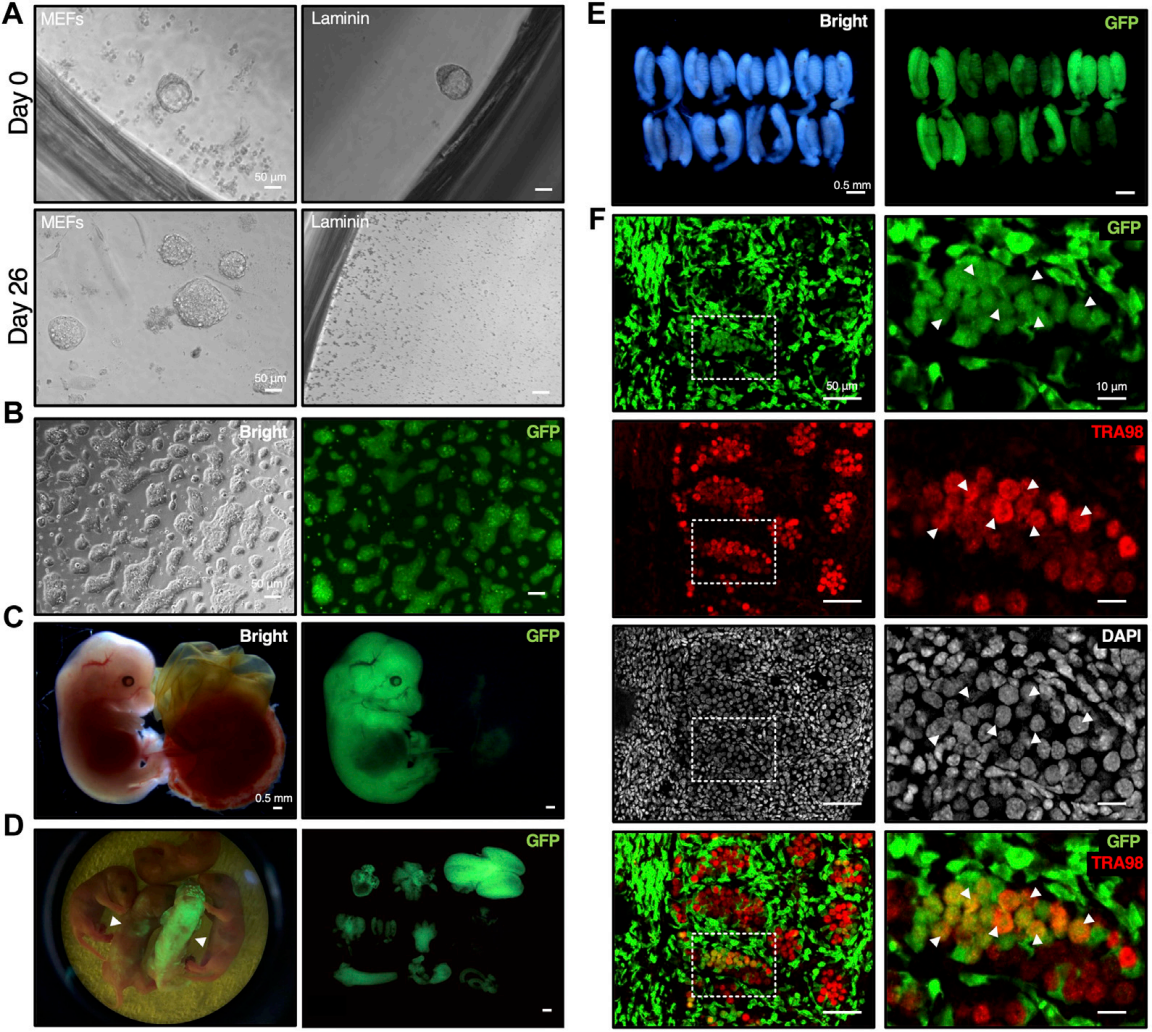
Establishment of mESCs from mouse blastocyst with DARP^2iLIF^ medium involved in chimera formation. **(A)** Time course of mESC lines establishment under DARP medium conditions. The E3.5 blastocysts derived from crossbred mice bearing the CAG-GFP transgene were cultured with DARP medium on MEF feeder cells or on the Laminin-coated plates (Day 0). In 2nd passage on day 26, the growing mESC colonies emerged on MEF feeder cells, but not on the Laminin-coated plates. **(B)** Representative images of the established mESCs under DARP medium condition (“DARP-ESCs” at passage 7). These cells were adapted onto the Laminin coated plates after establishment for serial passages. **(C)** Representative images of the chimeric embryos of DARP-ESCs. The chimeric mice isolated at E13.5 were generated by the aggregation of wild-type 8-cell embryos and DARP-ESCs. The contribution of DARP-ESCs is detectable by GFP signal. **(D)** Image of the chimera pups of DARP-ESCs (left) and those of isolated various tissues such as heart, liver, brain, lung, and intestine, etc., where DARP-ESC-derived cells are contributed (right). The white-filled arrowheads indicate chimera pups at 0 days of postnatal age contributed with GFP-expressing DARP-ESCs. **(E)** Representative images of the genital ridges of the chimeric embryos of DARP-ESCs at E13.5. **(F)** Immunofluorescence of the cryo-sectioned genital ridges from chimeric embryos of DARP-ESCs at E13.5. The white-filled arrowheads indicate the TRA98-positive gonadal germ cells derived from DARP-ESCs. Images on the right panels represent square dot lines in the images on the left panels at higher magnification. Nuclei were counterstained with DAPI. Merged images with GFP and TRA98 are shown on the bottom.

To determine the feasibility of establishing mESC lines using DARP medium, isolated mouse transgenic blastocysts which constitutively expressing *GFP* gene were cultured with DARP medium on feeder layer or on laminin-coated plates (Figure 5A). On laminin-coated plates, no colony formed even after a single passage (*n* = 32), but 9 out of 24 cultured blastocysts showed colonies on MEF feeder cells after serial passages (Figure 5A). Although the MEFs feeder layer was required for the early phase of mESC establishment from blastocysts using DARP medium, the established mESCs on MEF feeder cells could be adapted to feeder-free laminin-coated conditions through passages (Figure 5B).

The most reliable approach to verify the developmental potential of mESCs is to generate chimeric mice by blastocyst injection or embryo aggregation. Using GFP-positive DARP-mESCs in chimera formation assays by aggregation with 8-cell embryos, DARP-mESCs successfully contributed to the whole tissues in embryos at E13.5 and in newborn pups (Figures 5C, D). A contribution of DARP-mESCs to the germline was confirmed by the immunofluorescence analysis with the genital ridges of chimeric embryos at E13.5 (Figures 5E, F). These results demonstrated that DARP-mESCs indeed possess a naïve pluripotent state capable of contributing to germline chimera formation.

### The utility of DARP medium for the maintenance of pluripotency in the mouse primed state

**FIGURE 6:**
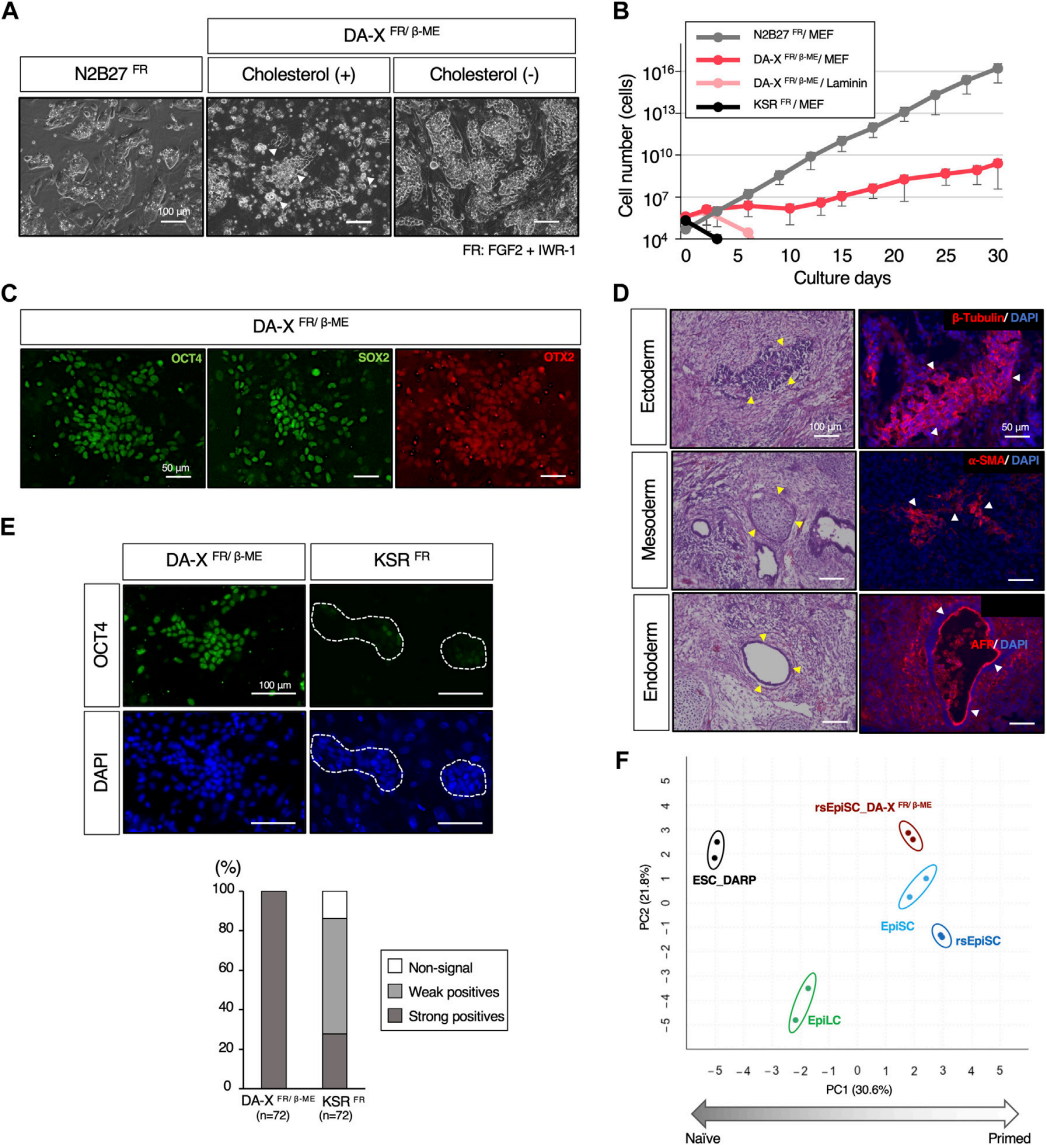
The maintenance of pluripotency in the mouse primed state with DA-X medium. **(A)** Representative images of colony morphology of cultured rsEpiSCs on MEF feeder cells under indicated conditions. Images were captured in indicated culture conditions. N2B27^FR^ medium on day 3 of passage 4, DA-X^FR/β−ME^ medium with cholesterol on day 3 of passage 1 and without cholesterol on day 3 of passage 4 The white-filled arrowheads indicate numerous dead cells. **(B)** The Growth curves of mouse rsEpiSCs for 30 days. The cells were cultured in each indicated condition. **(C)** Representative images of OCT4 and SOX2 (undifferentiated markers), and OTX2 (a primed marker) protein expression in the rsEpiSC colonies cultured in DA-X^FR/β−ME^ on MEF feeder cells at passage 5. **(D)** Histological analysis by HE stains and immunostaining with sections from teratomas generated in nude mice from rsEpiSC cultured in DA-X^FR/β−ME^ medium. The yellow-filled arrowheads show distinctive organizational tissues for neuroepithelium (ectoderm, top left), cartilage (mesoderm, middle left), and gland (endoderm, bottom left). The white-filled arrowheads indicate expression of characteristic marker protein, β-Tubulin for neuroepithelium (top right), α-SMA for smooth muscle (middle right), and AFP for glands (bottom right). Nuclei were counterstained with DAPI. **(E)** Comparison of the capacity to maintain undifferentiated state of rsEpiSCs between DA-X and KSR medium under FGF2 and IWR-1 supplementation. Representative immunofluorescence images showing strong (in DA-X^FR/β−ME^ medium) and weak expression (in KSR^FR^ medium) of OCT4 in rsEpiSC colonies cultured on day 3. Nuclei were counterstained with DAPI. Dotted lines in the images represent rsEpiSC colonies. The frequency of OCT4-positive colonies according to the signal intensity was calculated as a percentage of total nuclei. The total number of counted colonies is indicated in the graph. **(F)** Bioinformatic analysis of RNA-sequencing data with mouse pluripotent stem cells including those in primed state cultured in DA-X^FR/β−ME^ condition. (Left) Principal component analysis (PCA) visualization with differentially expressed genes (DEGs) among five groups (depicted with colored circles) showing distinct clustering according to their pluripotent states, naïve to primed. Replicates of the same cell lines and conditions are depicted with colored circles, ESC_DARP (black), EpiLC (green, published data set (Shirane et al., 2016)), EpiSC (light blue, published data set (Wu et al., 2015)), rsEpiSC (blue, published data set (Wu et al., 2015)), and rsEpiSC_ DA-X^FR/β-ME^ (brown).

A primed ground state of rsEpiSCs is captured by adding FGF2 and Wnt inhibitor, IWR-1 to N2B27 basal medium, N2B27^FR^, which is the diametrically opposite to a naïve ground state (Figure 6A) (Wu et al., 2015). To explore the extended applicability of the DA-X medium beyond the naïve state of mESCs, we attempted to culture mEpiSCs in the primed state. Unexpectedly, in contrast to mESCs, the addition of cholesterol to the DA-X-modified medium supplemented with FGF2, IWR-1, and β-ME (DA-X^FR/β−ME^) was extremely toxic to rsEpiSCs, indicating that the responsiveness to cholesterol varies between pluripotent states (Figure 6A). Although the growth rate in cholesterol-free DA-X^FR/β-ME^ was much slower than that in the N2B27^FR^, the cholesterol-free DA-X^FR/β-ME^ medium showed potential for long-term maintenance of rsEpiSCs with the support of MEF feeder cells (Figure 6B). The primed pluripotency of rsEpiSCs in the DA-X-modified medium was confirmed by immunofluorescence against OCT4 and SOX2 as pluripotent markers, and OTX2 as a primed marker (Figure 6C). In addition, the differentiation potential of rsEpiSCs in DA-X medium *in vivo* was confirmed by histological analysis of teratomas (Figure 6D).

Our results demonstrated that DA-X medium can support a broad spectrum of pluripotent stem cells. On the other hand, KSR-containing DMEM medium, which was the most efficient for naïve state of mESCs (Figure 2B), was unable to maintain rsEpiSCs after one passage even with FGF2 and IWR-1 (Figures 6B, E). Although only N2B27-based medium have been shown to be applicable to rsEpiSCs so far (Wu et al., 2015), DA-X^FR/β-ME^ medium provides an additional culture platform for rsEpiSC.

We then conducted RNA-sequencing analysis to explore whether the gene expression profiles of rsEpiSCs under DA-X^FR/β-ME^ medium are those in the primed state. The PCA of gene expression profiles revealed that rsEpiSCs cultured under DA-X^FR/β-ME^ clustered together with EpiSCs and rsEpiSCs cultured on N2B27^FR^ medium, suggesting that they share similar characteristics in the primed states (Figure 6F).

## Discussion

This study aimed to address the limitations of conventional serum-containing media and serum-free culture conditions based on commercially available supplements for mESCs and rsEpiSCs. These were achieved by formulating a chemically defined and compositionally disclosed serum-free culture medium based on the previously developed DA-X medium (Takii et al., 2022). The advance of the serum-free medium developed in this study facilitates the feasible comparison of results across different laboratories, since researchers will be able to obtain more consistent and reliable experimental results without having to rely on serum that varies between batches or expensive commercial supplements. The other advance is to facilitate mechanistic studies. By employing serum-free media with fully disclosed compositions and their concentrations provides researchers with a valuable tool to explore the underlying mechanisms of pluripotency and self-renewal in any stem cell (Wei et al., 2023). The flexibility to systematically modify the composition and concentration of the media allows the role of individual components, such as “cholesterol” as illustrated in this study or signaling pathways in governing stem cell behavior to be investigated. This knowledge deepens our understanding of stem cell biology and holds potential for the further development of novel culture techniques and therapeutic applications.

Despite extensive research on the role of cholesterol in cell proliferation of various cell types, including cancer cells (Maja and Tyteca, 2022) and somatic stem cells (Oguro, 2019), little is known about cholesterol’s role in mESC. The results of this study are the first to demonstrate that cholesterol is important for the proliferation and maintenance of mESCs, just as cholesterol plays the significant roles in self-renewal and long-term survival of other stem cells. Cholesterol plays a critical role in the formation and stability of lipid rafts (Silvius, 2003), which are cholesterol-enriched microdomains within the cell membrane that serve as platforms for various signaling molecules (Nishihara, 2018). Based on the results of this study, mESC cell membrane may promote the formation of stable lipid rafts if cholesterol concentrations are increased, resulting in enhanced signal transduction of the other growth factors or cytokines (Maja and Tyteca, 2022). Despite being two strong candidates (Lee et al., 2010; Karouzaki et al., 2021; Ogawa et al., 2006; Sheng et al., 2014), cholesterol did not affect LIF and Wnt signaling (Supplementary Figures S2A,B). Further research is needed to fully understand the precise mechanisms by which cholesterol affects mESC proliferation, but this knowledge has the potential to shed light on the molecular mechanisms underlying mESC self-renewal and the regulation of cell cycle progression.

The utility of DA-X medium was also explored for the maintenance of pluripotency in mouse rsEpiSCs, which are in primed ground state (Figure 6). Successful long-term cultivation of mouse rsEpiSCs was achieved in DA-X^FR/β-ME^, indicating the potential broader application of DA-X medium as a basal medium even for maintaining pluripotency regardless of their state (Wei et al., 2023). By utilizing the DA-X medium, we found that cholesterol significantly reduced the viability of rsEpiSCs (Figure 6A). Although the precise mechanism explaining the cytotoxic response of rsEpiSCs to cholesterol remains unclear, there may be significant differences in cholesterol metabolism between naïve and primed pluripotent states in mice, which could influence cell survival and proliferation. Various cellular metabolic distinctions, including energy, amino acid, and fatty acid metabolism, between naïve and primed pluripotent stem cells in mice and humans have been elucidated (Zhou et al., 2012). Further research is needed to fully comprehend the cholesterol metabolism underlying the cytotoxic response of rsEpiSCs to cholesterol (Song et al., 2021) and to search for substances to supply to DA-X^FR/β-ME^ medium to achieve cell growth comparable to that of N2B27 medium (Figure 6B). Thus, one of the most promising applications of our DA-X-based medium is its ability to fine-tune the components based on the disclosed medium composition, regardless of pluripotency status (naïve or primed). These findings will offer valuable insights into the specific components and culture conditions required to control the properties of mouse pluripotent stem cells. In the future, it will be essential to verify the suitability of these media for other animal species besides mice.

## Data Availability

The datasets presented in this study are available in online repositories. The data have been deposited to BioProject, accession number PRJDB17732.

## Supplementary material

SUPPLEMENTARY FIGURE S1 The minimum concentraion of laminin required for robust growth of mESCs. Representative images of mESCs cultured in DARP medium on different concentration of laminin, captured on day 4. The concentration of 0.5 μg/cm^2^ is the manufacture’s recommended concentration.

SUPPLEMENTARY FIGURE S2 The effects of cholesterol supplementation in mESC. **(A)** Western blot analyses for the expression of AKT, phosphorylated AKT, STAT3, phosphorylated STAT3 in mESCs cultured with or without cholesterol in DA-X^2iLIF/β-ME^ medium. β-Actin was used as loading control. **(B)** Representative images of R26-WntVis mESCs with or without cholesterol in DA-X^PD03/LIF/β−ME^ medium (described in Supplementary Table S1), in which CHIR99021 (GSK-3 inhibitor/ Wnt activator) was withdrawal. The activity of the Wnt signaling pathway monitored by the EGFP expression (Wnt-EGFP) with or without cholesterol was analyzed by imaging for protein (left) and quantitative PCR analysis (right) on day 3. Error bars indicate s.d. (*n* = 3, biological replicates).

SUPPLEMENTARY FIGURE S3 The effect of NEAA supplementation on mESC growth. The growth curves for comparison of mESC growth with or without NEAA in DARP medium. Error bars indicate s.d. (*n* = 3, biological replicates).

SUPPLEMENTARY FIGURE S4 The chromosomal stability of mESCs in DARP medium. Chromosomal karyotyping performed with Giemsa banding of mESCs cultured in the indicated conditions at more than 20 passages by measuring the number of condensed chromosomes per cell. Error bars indicate s.d. (*n* = 141, the number of measured cells respectively).

SUPPLEMENTARY TABLE S1 Medium components used in this study.
