# Supplementary figures and images for: Development of a chemically disclosed serum-free medium for mouse pluripotent stem cells
Source: Front Bioeng Biotechnol. 2024 May 15;12:1390386. doi: 10.3389/fbioe.2024.1390386 (PMC11134454; doi:10.3389/fbioe.2024.1390386)

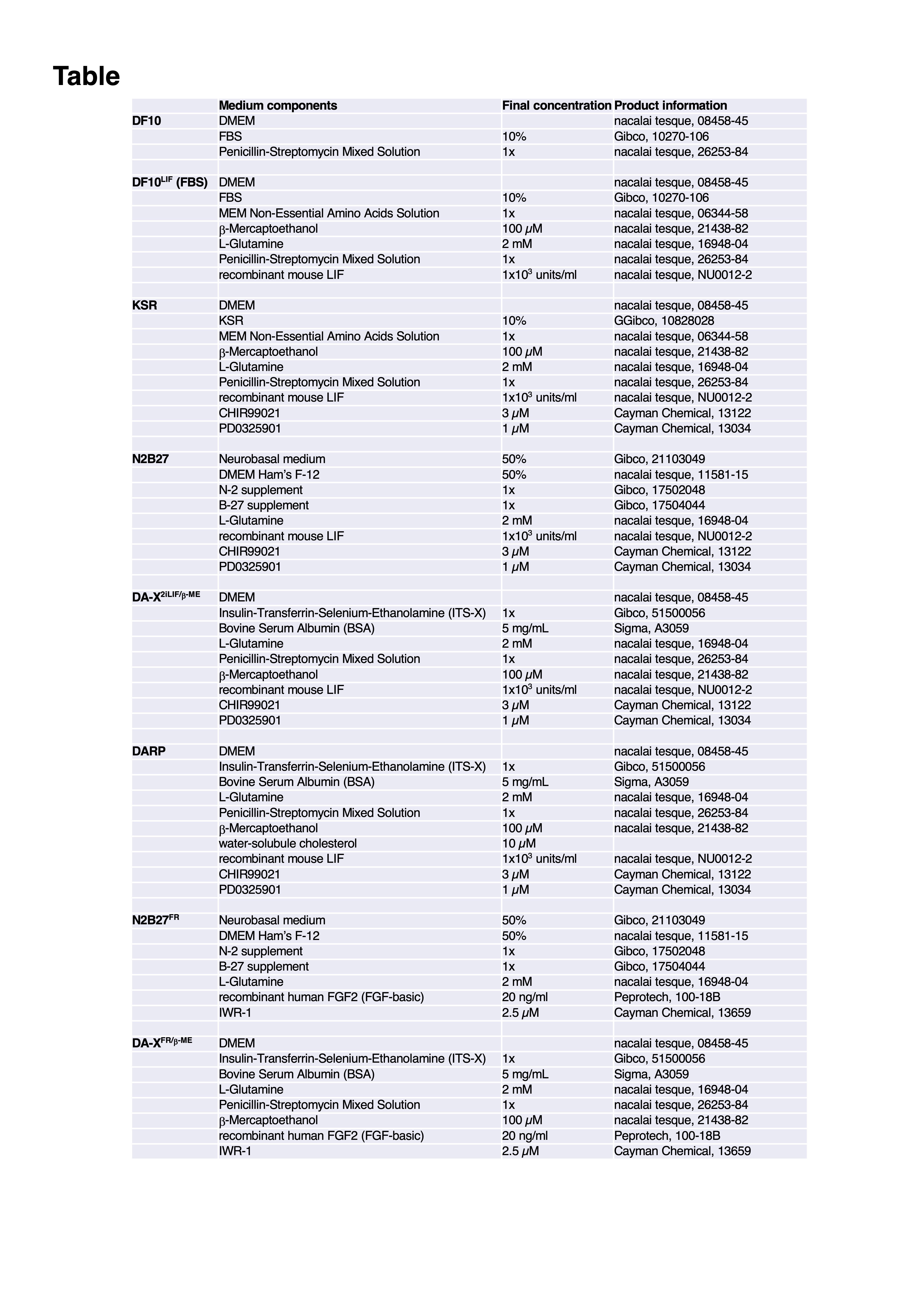

Supplement: Supplementary file 1 [file Image5.jpg]

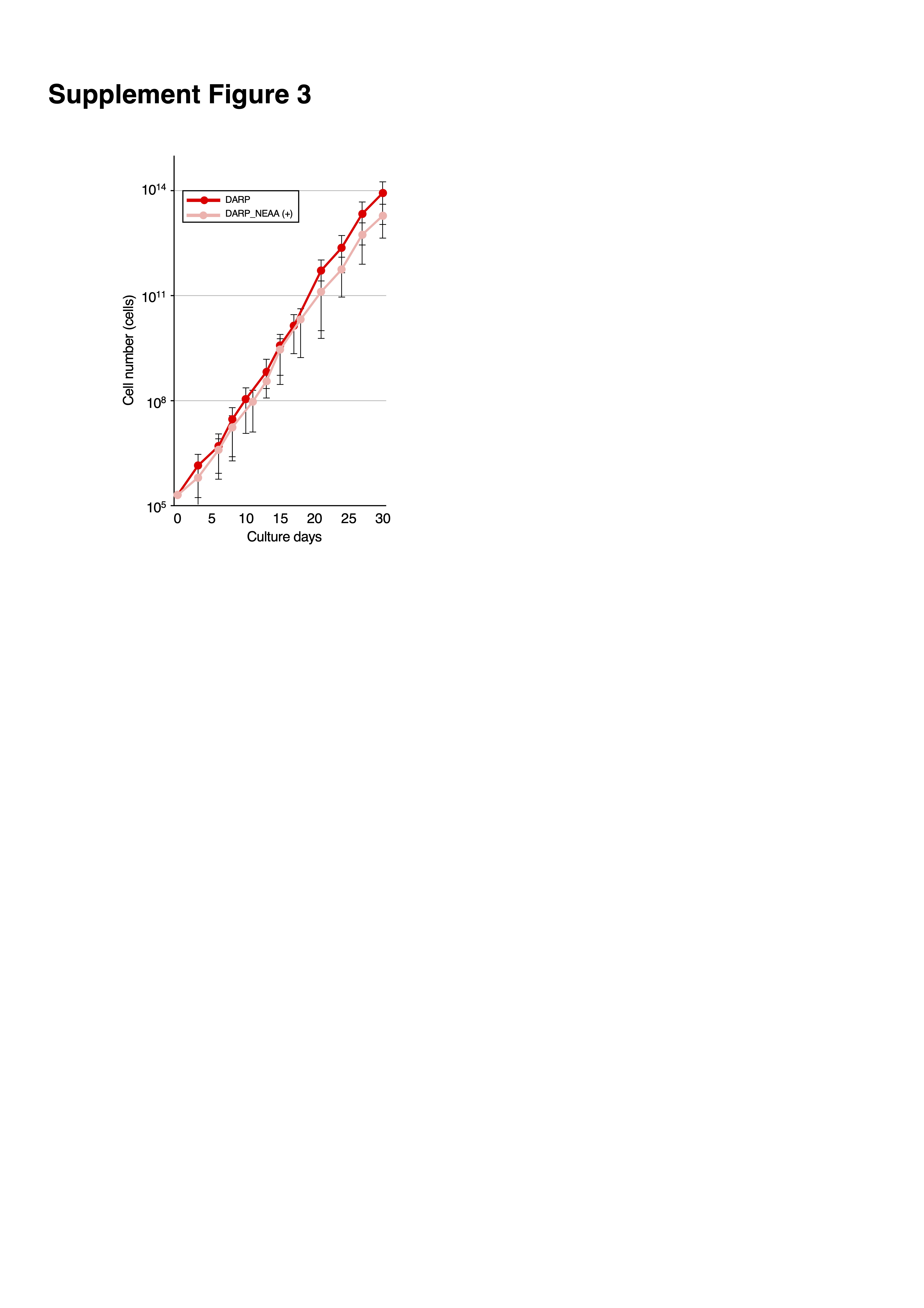

Supplement: Supplementary file 2 [file Image3.jpg]

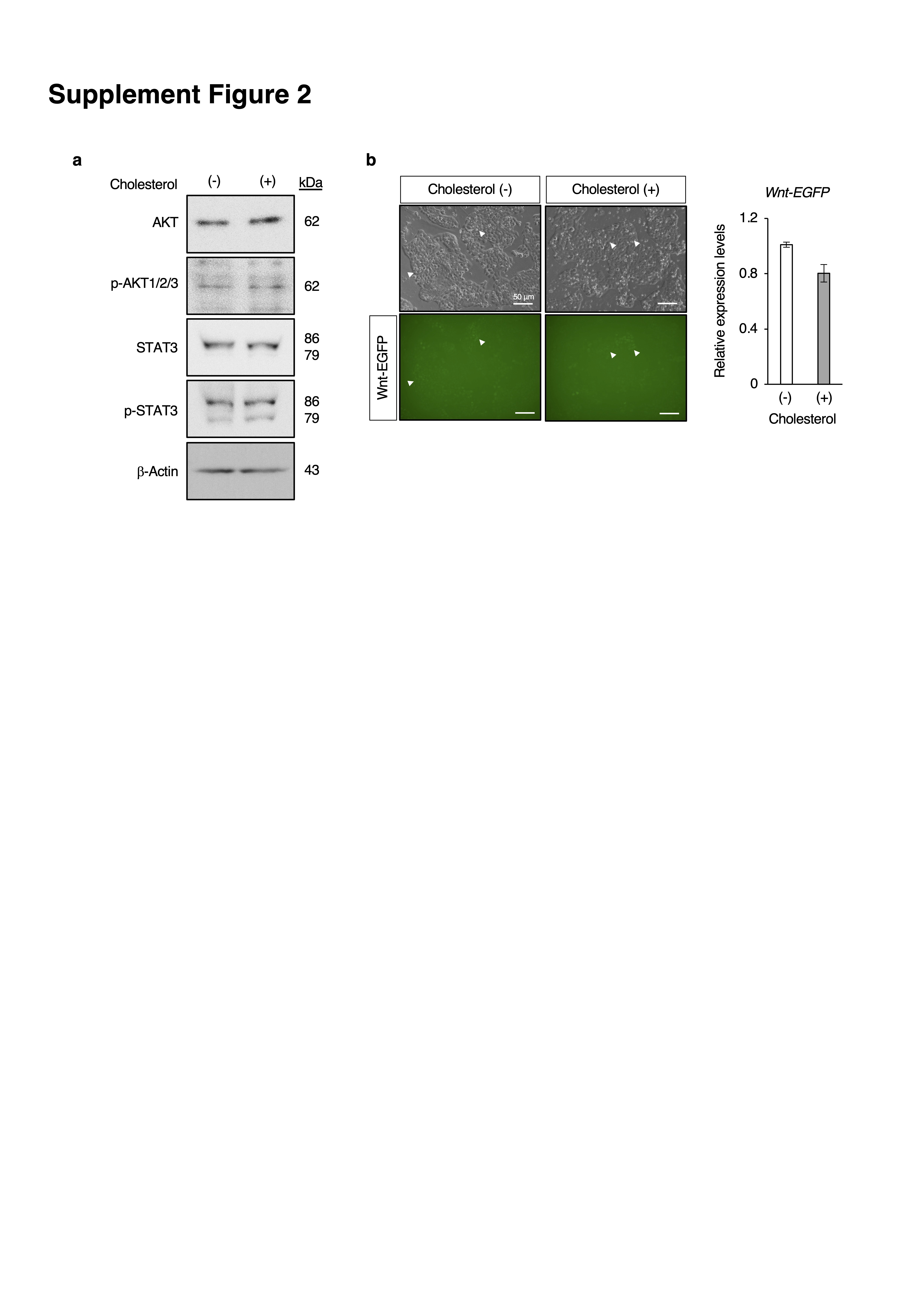

Supplement: Supplementary file 3 [file Image2.jpg]

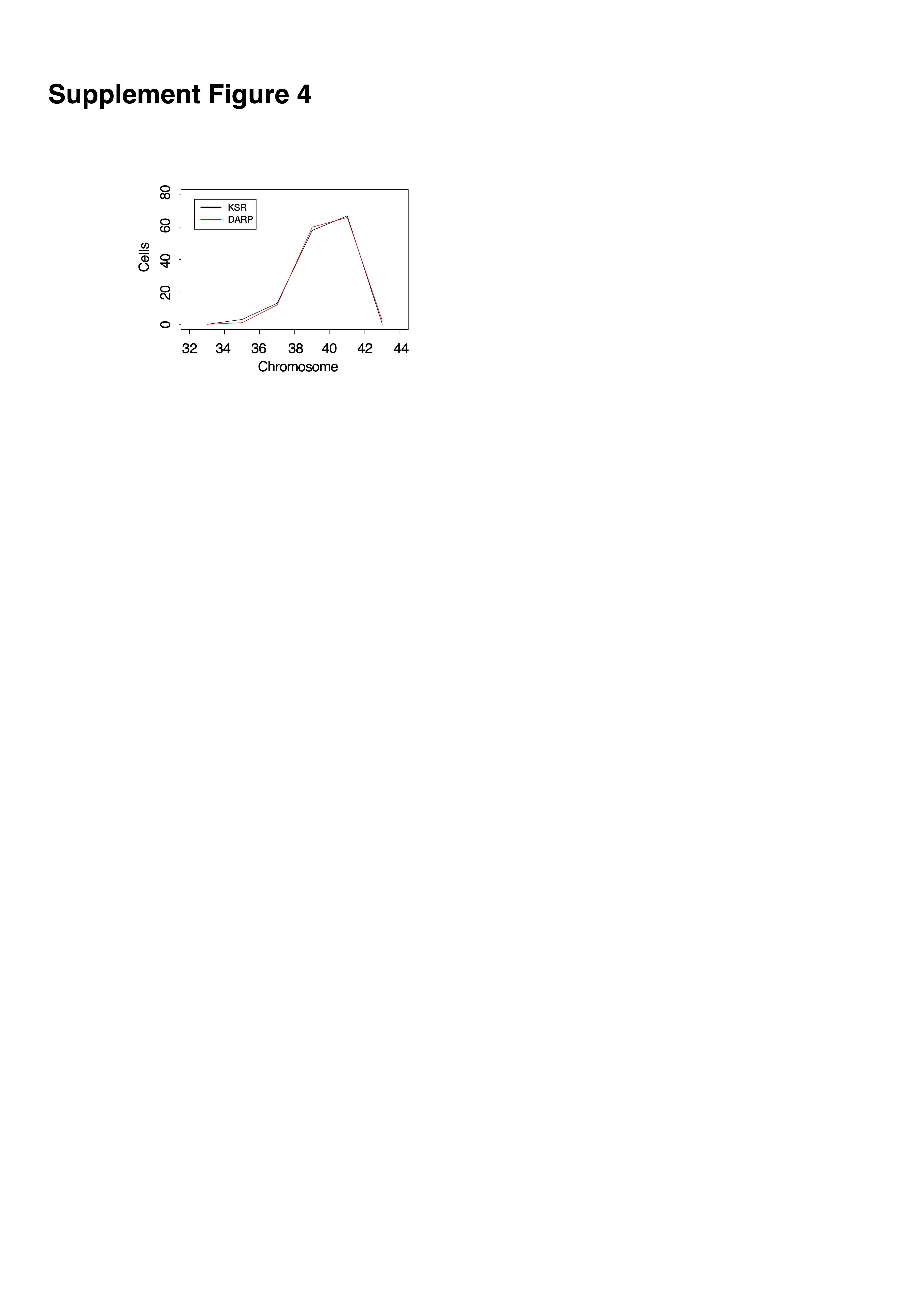

Supplement: Supplementary file 4 [file Image4.jpg]

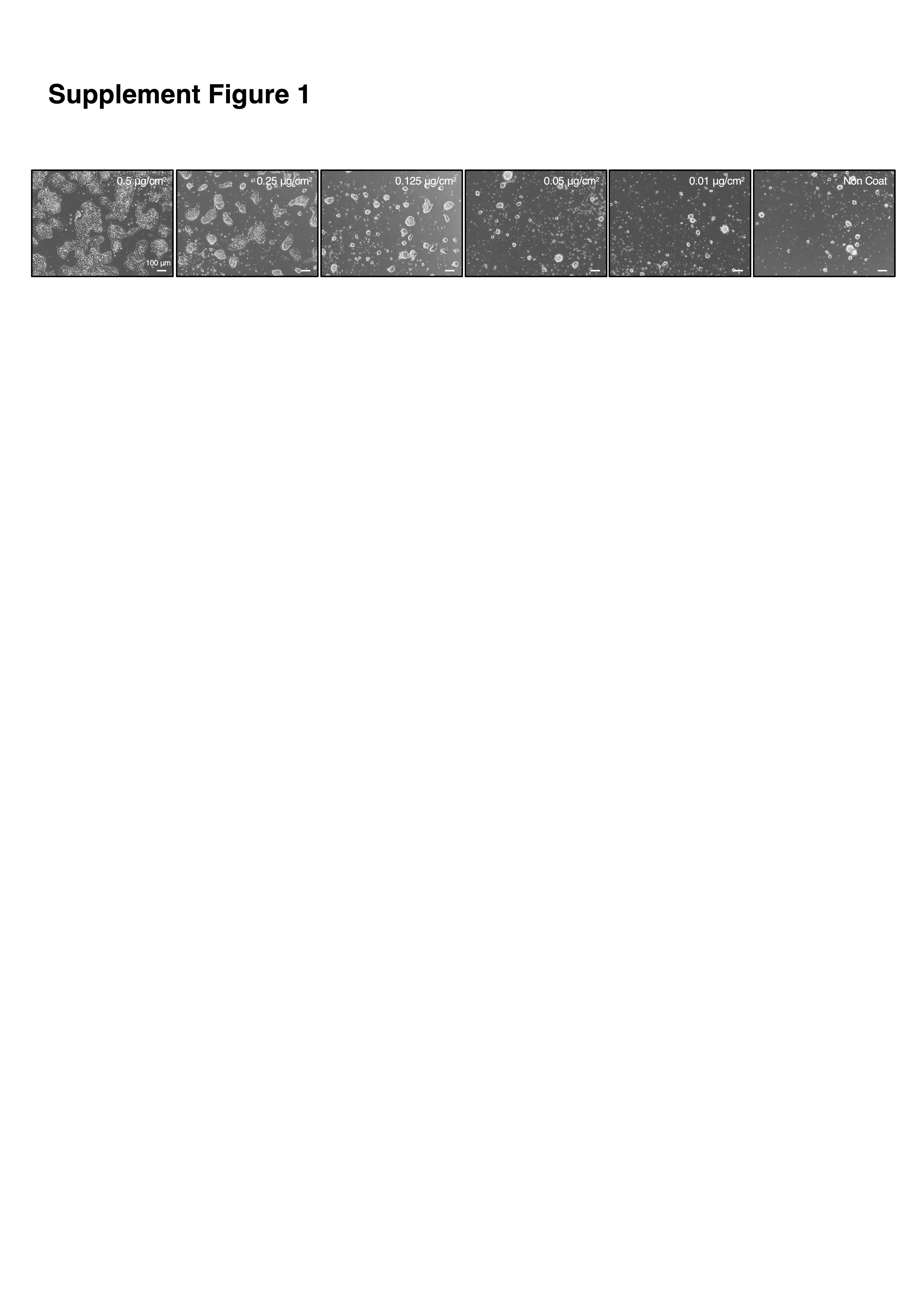

Supplement: Supplementary file 5 [file Image1.jpg]
